# Host Lung Environment Limits Aspergillus fumigatus Germination through an SskA-Dependent Signaling Response

**DOI:** 10.1128/msphere.00922-21

**Published:** 2021-12-08

**Authors:** Marina E. Kirkland, McKenzie Stannard, Caitlin H. Kowalski, Dallas Mould, Alayna Caffrey-Carr, Rachel M. Temple, Brandon S. Ross, Lotus A. Lofgren, Jason E. Stajich, Robert A. Cramer, Joshua J. Obar

**Affiliations:** a Geisel School of Medicine at Dartmouth, Department of Microbiology & Immunology, Lebanon, New Hampshire, USA; b Montana State University, Department of Microbiology & Immunology, Bozeman, Montana, USA; c Department of Microbiology & Plant Pathology, University of California Riverside, Riverside, California, USA; d Institute for Integrative Genome Biology, University of California Riverside, Riverside, California, USA; University of Georgia

**Keywords:** *Aspergillus fumigatus*, aspergillosis, germination, inflammation

## Abstract

Aspergillus fumigatus isolates display significant heterogeneity in growth, virulence, pathology, and inflammatory potential in multiple murine models of invasive aspergillosis. Previous studies have linked the initial germination of a fungal isolate in the airways to the inflammatory and pathological potential, but the mechanism(s) regulating A. fumigatus germination in the airways is unresolved. To explore the genetic basis for divergent germination phenotypes, we utilized a serial passaging strategy in which we cultured a slow germinating strain (AF293) in a murine-lung-based medium for multiple generations. Through this serial passaging approach, a strain emerged with an increased germination rate that induces more inflammation than the parental strain (herein named LH-EVOL for lung homogenate evolved). We identified a potential loss-of-function allele of *Afu5g08390* (*sskA*) in the LH-EVOL strain. The LH-EVOL strain had a decreased ability to induce the SakA-dependent stress pathway, similar to AF293 Δ*sskA* and CEA10. In support of the whole-genome variant analyses, *sskA*, *sakA*, or *mpkC* loss-of-function strains in the AF293 parental strain increased germination both *in vitro* and *in vivo*. Since the airway surface liquid of the lungs contains low glucose levels, the relationship of low glucose concentration on germination of these mutant AF293 strains was examined; interestingly, in low glucose conditions, the *sakA* pathway mutants exhibited an enhanced germination rate. In conclusion, A. fumigatus germination in the airways is regulated by SskA through the SakA mitogen-activated protein kinase (MAPK) pathway and drives enhanced disease initiation and inflammation in the lungs.

**IMPORTANCE** Aspergillus fumigatus is an important human fungal pathogen particularly in immunocompromised individuals. Initiation of growth by A. fumigatus in the lung is important for its pathogenicity in murine models. However, our understanding of what regulates fungal germination in the lung environment is lacking. Through a serial passage experiment using lung-based medium, we identified a new strain of A. fumigatus that has increased germination potential and inflammation in the lungs. Using this serially passaged strain, we found it had a decreased ability to mediate signaling through the osmotic stress response pathway. This finding was confirmed using genetic null mutants demonstrating that the osmotic stress response pathway is critical for regulating growth in the murine lungs. Our results contribute to the understanding of A. fumigatus adaptation and growth in the host lung environment.

## INTRODUCTION

Aspergillus fumigatus is a ubiquitous mold essential for decomposition of organic material that can easily become airborne. Infection is established in the respiratory tract by inhalation of dormant conidia which are roughly 2 μm in diameter. Their small size facilitates embedment into tissues if those resting conidia are not removed through mucociliary action of respiratory epithelial cells. If allowed sufficient time, A. fumigatus conidia will begin to germinate, which initiates with osmotic swelling and then polarized growth of the germ tube. At this point, a healthy immune system can detect pathogen-associated molecular patterns (PAMPs) on the cell surfaces of swollen or germinated conidia, clearing the swollen conidia before disease can be established. Should this occur in an immunocompromised host, germination can go unchecked, resulting in fungal biofilm formation and tissue invasion establishing severe disease (1–3).

Previous work has shown a correlation between germination and pathogenicity of A. fumigatus in a strain-dependent manner in a model of bronchopneumonia (4). Between clinical isolates AF293 and CEA10, CEA10 exhibits faster germination both *in vitro* and *in vivo* in murine lung airways (4), as well as in a zebrafish infection model (5). This increased germination corresponds with greater tissue damage and greater inflammation following CEA10 challenge compared to AF293 challenge (4–6). However, conditions and genetic pathways that regulate the germination rate for each strain in the lung microenvironment remain unresolved.

For germination to occur, conidia must first sense whether their environment has sufficient nutrient availability and the presence of potential stressors. In Aspergillus nidulans, the presence of glucose was sufficient to induce germination of conidia (7), while in A. fumigatus glucose in the presence of water and oxygen to drive cellular respiration was necessary for conidial germination (8). The germination process is regulated by both Ras signaling (9) and G-protein signaling through the cyclic AMP (cAMP)-protein kinase A (PKA) signaling pathway (8–10). Additionally, calcium signaling through the calcineurin pathway and the CrzA transcription factor is needed for A. fumigatus germination (11, 12). Further, in A. fumigatus, nitrogen sources, such as ammonium chloride or proline, drive more robust germination in a SakA-regulated manner (13). Once conidia begin to germinate, they begin an isotropic growth phase which modifies the composition of their cell walls, subjecting conidia to detection by a host’s immune system. Polarized growth follows, as swollen conidia develop into germlings and then hyphae before establishing a complex network called mycelia (1).

The lung itself has evolved to interact constantly with an onslaught of foreign particulate matter and microbes in the air. Lung airways are a low-nutrient environment: airway surface liquid (ASL) glucose concentrations in a healthy adult are approximately 0.4 mM, which is about 10-fold less than in the plasma (14). Reduced nutrient availability is thought to limit microbial growth in the lung environment. In addition, the ASL facilitates the first-line defense of airway epithelial cells by providing mucous and water secretions which assist in mucociliary clearance (14–16). The ASL can become dysregulated in chronic disease states brought on by asthma (17, 18), chronic obstructive pulmonary disease (COPD) (18), cystic fibrosis (19), and type II diabetes (19–21), resulting in increased glucose levels (21), all of which have increased risk for developing aspergillosis (22–25).

In this study, we explored the molecular mechanisms regulating the germination of A. fumigatus in the context of the murine lung environment. To do this, we utilized a serial passaging approach by culturing the slow germinating AF293 strain in lung homogenate medium and selecting for increased germination after multiple generations. A novel AF293-derived strain, LH-EVOL (for lung homogenate evolved), was selected and found to germinate quicker in the murine lung airways compared to the parental AF293 strain, resulting in a stronger proinflammatory interleukin 1α (IL-1α)-dependent immune response. Whole-genome sequencing of LH-EVOL revealed conserved mutations in *Afu5g08390* (*sskA*), which is part of the SakA mitogen-activated protein kinase (MAPK) stress pathway (26). *In vitro* and *in vivo* growth kinetics of an AF293 Δ*sskA* mutant recapitulated the quick germinating phenotype of LH-EVOL. Interestingly, this rapid germination phenotype was further accelerated *in vitro* under low glucose conditions, which more closely resemble the concentration of glucose found in the lung environment. Taken together, the results of this study further support previous observations that germination rate is a key factor in determining the qualitative and quantitative inflammatory immune response to A. fumigatus strains in the murine lung and identifies the SakA pathway as a key mediator of the host lung environment.

## RESULTS

### *In vitro* serial passaging of AF293 in lung homogenate medium leads to a strain with increased airway growth.

**FIG 1:**
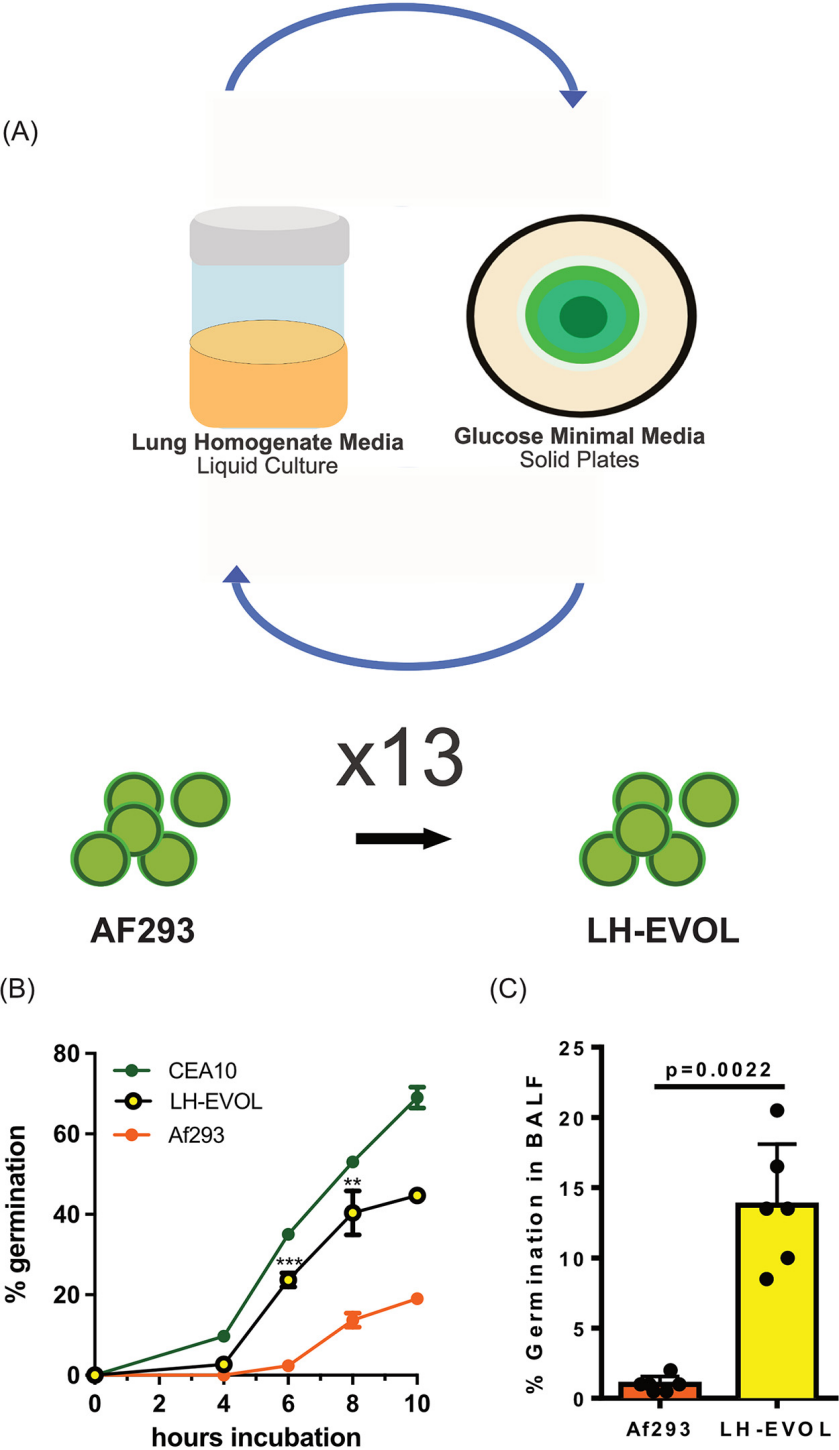
Lung evolved (LH-EVOL) A. fumigatus demonstrates increased *in vitro* and *in vivo* germination kinetics. (A) LH-EVOL strain was generated through serial passaging of the AF293 strain 13 times in lung homogenate medium. (B) Lung tissue from C57BL/6J mice was homogenized in PBS and used as the medium for *in vitro* germination assays. Percent germination over time in lung homogenate media of strain AF293, CEA10, or LH-EVOL was quantified every 2 h by microscopically counting the numbers of conidia and germlings. Data are represented as the percentage of fungal matter that germinated. Data are representative of at least two independent experiments consisting of three biological replicates per group. Each symbol represents the group mean ± 1 standard error of the mean (error bar). (C) C57BL/6J mice were challenged with 4 × 10^7^ conidia of strain AF293 or LH-EVOL. At 12 h postinfection (hpi), mice were euthanized, and BALF samples were collected to quantify germination in the airways. Data are representative of results from two independent experiments with five mice per group. In panels B and C, statistical significance was assessed between strains LH-EVOL and AF293 using a Mann-Whitney U test. **, *P* value of ≤0.01; ***, *P* value of ≤0.001.

Previously, we had established that strains of Aspergillus fumigatus, in the context of the lung environment, can be grouped into fast and slow germinators that induce markedly different pathologies (4, 27). Notably, fast germinating strains, such as CEA10, induce extensive loss of alveoli and destruction of the parenchyma with substantial hemorrhaging compared to slow germinating strains, such as AF293. To identify the potential molecular pathways responsible for the quick germination phenotype within the lung environment by A. fumigatus, we utilized an experimental serial passage approach, which enables us to identify potential fungal regulators of growth in the lung microenvironment without knowing the exact nutrients that are biologically available. Specifically, the slow germinating AF293 strain was serially cultured in lung homogenate medium for 8 h to initiate germination and then plated on 1% glucose minimal medium (GMM) (see Materials and Methods) plates for 3 days before collecting spores for cataloging and the next passage (Fig. 1A). After the strain was passaged 13 times, we identified a novel AF293-based strain, referred to as LH-EVOL, that was able to germinate rapidly in lung homogenate medium (Fig. 1B). The germination kinetics of the LH-EVOL strain in lung homogenate medium was significantly faster than that of its parental AF293 strain, while having similar germination kinetics to the CEA10 strain in this *in vitro* assay (Fig. 1B). To determine whether the LH-EVOL strain also has an increased germination rate during initial airway challenge of mice, C57BL/6J mice were inoculated with 4 × 10^7^ conidia of either parental strain AF293 or strain LH-EVOL. After challenge, significantly more germination by LH-EVOL within the bronchoalveolar lavage fluid (BALF) 12 h postinoculation was observed compared to its parental AF293 strain (Fig. 1C). Thus, our *in vitro* serial passaging experimental conditions drove the development of a novel AF293-based strain that can rapidly germinate within the lung airways of mice.

### Comparison of pulmonary inflammatory response induced following AF293 and LH-EVOL challenge in the murine lung.

**FIG 2:**
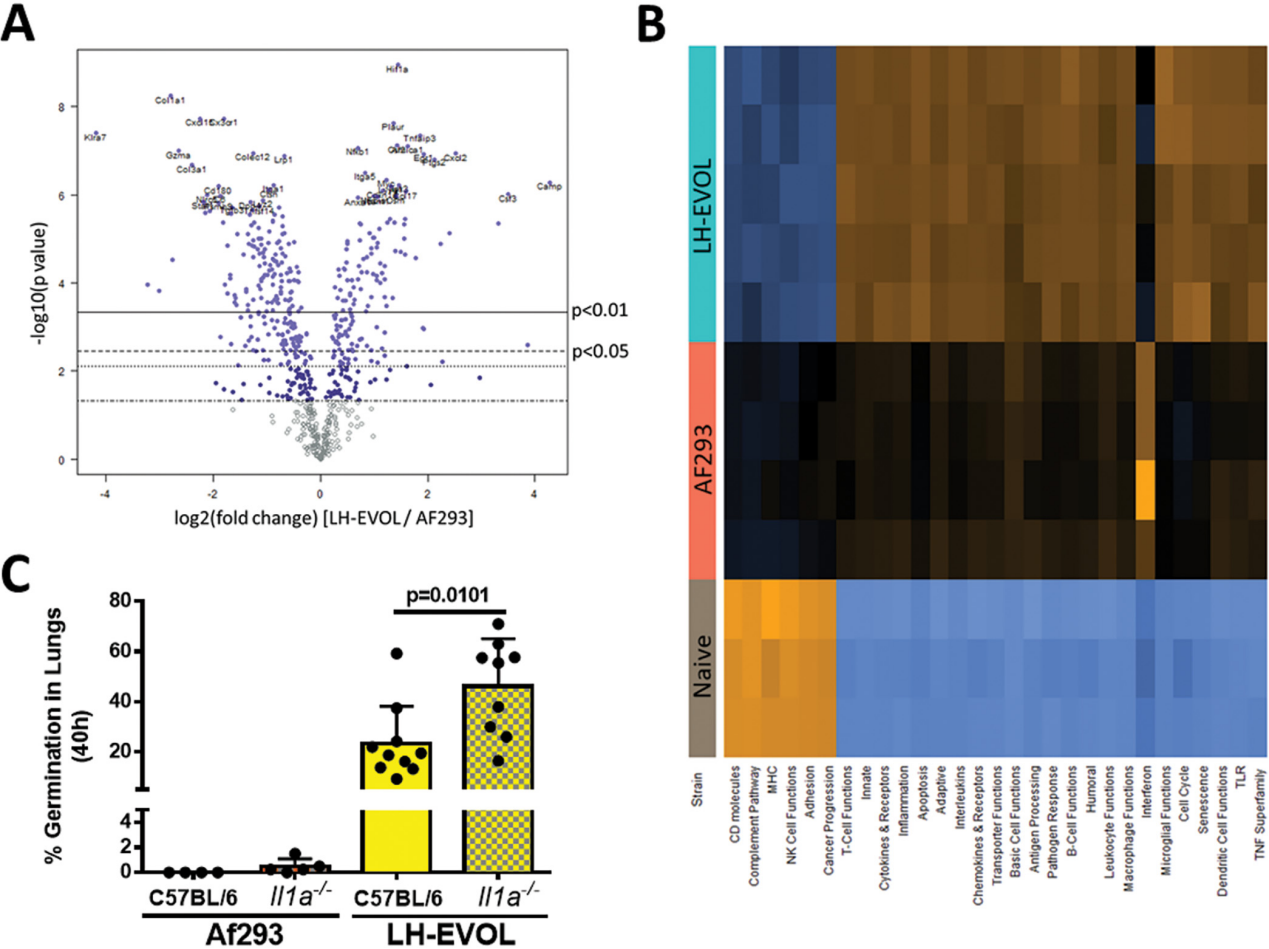
Differential pulmonary inflammatory gene expression and immune response after strain AF293 and LH-EVOL challenge. C57BL/6J mice were inoculated with either PBS or 4 × 10^7^ AF293 or LH-EVOL conidia and euthanized at 40 h postinoculation. Total mRNA was extracted from the whole lung, and gene expression was analyzed by NanoString nCounter PanCancer Immune Profiling panel. (A) Volcano plot showing the distribution of fold changes in gene expression in mice challenged with strain LH-EVOL compared to mice challenged with strain AF293. (B) Pathway signature of mice challenged with either PBS, AF293, or LH-EVOL. Orange color indicates a pathway is enriched, while blue color indicates a pathway is decreased. (C) C57BL/6J and *Il1a^−/−^* mice were inoculated with either 4 × 10^7^ conidia of either AF293 or LH-EVOL. At 40 h postchallenge, mice were euthanized, and lungs were saved for histological analysis. Formalin-fixed lungs were paraffin embedded, sectioned, and stained with GMS for microscopic analysis of fungal germination. Data are pooled from two independent experiments. Statistical significance was determined using a Mann-Whitney U test.

Given the different germination rates of the AF293 and LH-EVOL strains *in vivo*, we next analyzed the inflammatory response induced in the lung after challenge with each respective strain. Total RNA was extracted from whole lungs of C57BL/6J mice challenged 40 h prior with either AF293 or LH-EVOL. Changes in mRNA levels of immune-related genes were analyzed using NanoString nCounter technology. We observed strikingly different overall changes in mRNA levels in phosphate-buffered saline (PBS)-challenged animals compared to the two fungal strains (see Data Sets S1 and S2 in the supplemental material). Importantly, mRNA levels of 57 genes were significantly increased more than twofold following LH-EVOL challenge compared with AF293 challenge, whereas 65 genes were significantly increased more than twofold following AF293 challenge compared with LH-EVOL challenge (Fig. 2A and Data Set S3). When we examined differential gene expression and pathway analysis using nSolver Advanced Analysis software, we noted a dramatic signature related to interferon (IFN) signaling following AF293 challenge (Fig. 2B). This increased IFN signaling signature is evidenced by significant increases in *Cxcl9*, *Cxcl10*, *Stat1*, *Zbp1*, *Irf7*, *Isg15*, and *Ifit3* levels (Table 1). It has been established that early antifungal immune responses to conidia will induce expression of beta interferon (IFN-β) and CXCL10 (chemokine [C-X-C motif] ligand 10) (28); therefore, increased IFN signaling may occur in AF293 due to slower germination. In contrast, there were dramatic increases across several pathways associated with innate immunity and cytokine/chemokine signaling following LH-EVOL challenge relative to AF293 challenge (Fig. 2B). Increased mRNA expression levels of proinflammatory cytokines, including *Il1a*, *Il6*, *Csf2*, *Cxcl1*, *Cxcl2*, and *Cxcl5* and expression levels of the transcription factors *Nfkb1* and *Hif1a* were elevated following LH-EVOL challenge (Table 2).

**TABLE 1:** Select gene transcripts upregulated following AF293 challenge compared to LH-EVOL challenge

Gene	Fold change (log_2_)	*P* value
*Cxcl9*	2.92	0.0155
*Cxcl10*	2.68	0.000138
*Gzma*	2.57	0.000536
*Stat1*	2.11	0.000174
*Angpt1*	2.07	0.00212
*Nlrc5*	2.04	0.00163
*Zbp1*	1.73	0.00243
*Ddx60*	1.66	0.00553
*Gbp5*	1.52	0.0273
*Irf7*	1.35	0.00715
*Isg15*	1.32	0.0182
*Ifit3*	1.27	0.0357
*Ddx58*	0.664	0.0166
*Ifih1*	0.296	n.s.

**TABLE 2:** Select gene transcripts upregulated following LH-EVOL challenge compared to AF293 challenge

Gene	Fold change (log_2_)	*P* value
*Camp*	4.34	0.000918
*Csf3*	3.57	0.000115
*Cxcl2*	2.59	0.000423
*Cxcl1*	2.47	0.000918
*Il6*	2.32	0.00212
*Ptgs2*	2.20	0.000369
*Hif1a*	1.51	0.000249
*Csf2*	1.51	0.000369
*Cxcl5*	1.42	0.00515
*Il12a*	1.23	0.00442
*Nfkbia*	1.08	0.000139
*Il1a*	1.06	0.000369
*Nfkb1*	0.77	0.000369
*Il1b*	0.66	0.00139

10.1128/msphere.00922-21.8 DATA SET S1 Differential gene expression in the lungs between PBS- and AF293-challenged C57BL/6J mice at 40 h postchallenge as assessed by NanoString analysis. Download Data Set S1, XLSX file, 0.1 MB. Copyright © 2021 Kirkland et al. 2021 Kirkland et al. https://creativecommons.org/licenses/by/4.0/ This content is distributed under the terms of the Creative Commons Attribution 4.0 International license.

10.1128/msphere.00922-21.9 DATA SET S2 Differential gene expression in the lungs between PBS- and LH-EVOL-challenged C57BL/6J mice at 40 h postchallenge as assessed by NanoString analysis. Download Data Set S2, XLSX file, 0.1 MB. Copyright © 2021 Kirkland et al. 2021 Kirkland et al. https://creativecommons.org/licenses/by/4.0/ This content is distributed under the terms of the Creative Commons Attribution 4.0 International license.

10.1128/msphere.00922-21.10 DATA SET S3 Differential gene expression in the lungs between AF293- and LH-EVOL-challenged C57BL/6J mice at 40 h postchallenge as assessed by NanoString analysis. Download Data Set S3, XLSX file, 0.1 MB. Copyright © 2021 Kirkland et al. 2021 Kirkland et al. https://creativecommons.org/licenses/by/4.0/ This content is distributed under the terms of the Creative Commons Attribution 4.0 International license.

The marked elevation of *Il1a* transcripts observed following LH-EVOL challenge fits with our previous observation that IL-1α is essential for host resistance against highly virulent isolates of A. fumigatus that can rapidly germinate in the lung airways (4, 29). To test the importance of IL-1α in host resistance against the LH-EVOL strain, C57BL/6J mice and *Il1a* ^−/−^ mice were challenged with either strain AF293 or LH-EVOL. At 40 h postchallenge, lungs were collected for histological analysis to determine fungal germination. We found that not only did C57BL/6J and IL-1α-deficient mice challenged with LH-EVOL have significantly greater fungal germination than mice challenged with AF293 (Fig. 2C), but IL-1α-deficient mice challenged with LH-EVOL also had significantly more fungal germination than C57BL/6J mice challenged with LH-EVOL (Fig. 2C). Taken together, these data reveal that the rapid germinating LH-EVOL strain is markedly more inflammatory.

### Genomic analysis of LH-EVOL identifies a potential truncation mutation within *Afu5g08390* (*sskA*).

**FIG 3:**
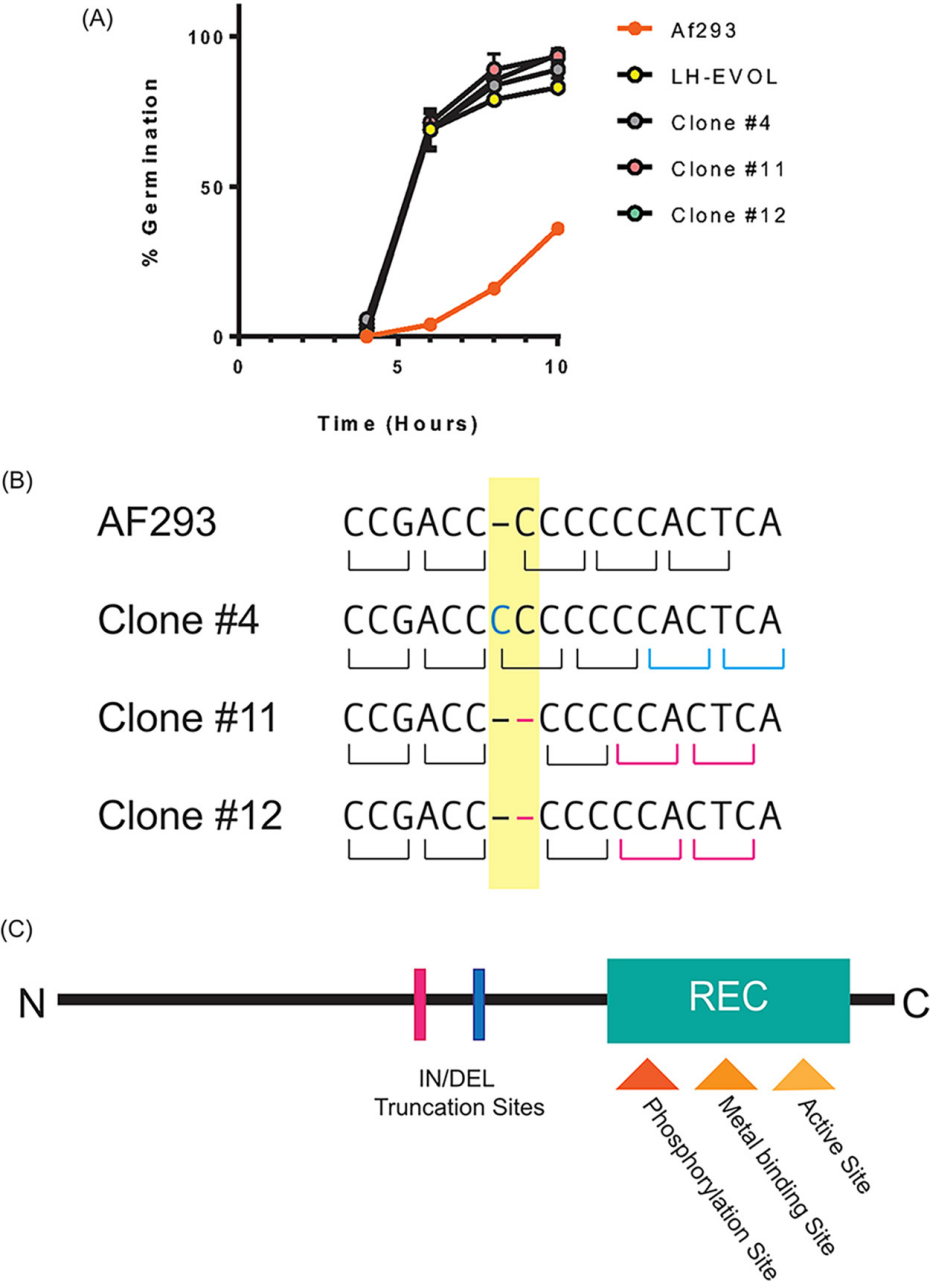
Identification of indel mutations in the *Afu5g08390* (*sskA*) gene in the LH-EVOL strain of A. fumigatus. (A) Percent germination over time in lung homogenate media of strain AF293, the bulk LH-EVOL strain, or individual LH-EVOL clones (clone 4, clone 11, or clone 12). Data are representative of at least two independent experiments consisting of three biological replicates per group. Each symbol represents the group mean ± 1 standard error of the mean. (B) DNA sequencing of LH-EVOL clones 4, 11, and 12 revealed indel mutations in the *Afu5g08390* (*sskA*) gene. (C) Based on the predicted amino acid changes due to the frameshift caused by these indel mutations, it is predicted that a premature stop codon causes the truncation of Ska prior to its REC domain in the LH-EVOL strains.

Selective serial culturing of an organism will often result in genetic modifications as the organism responds to a given environmental pressure, such as growth in the lung microenvironment without direct *a priori* knowledge about the exact nutrients that are biologically available for fungal growth. Therefore, we asked what genomic changes arose in strain LH-EVOL to determine the genetic basis for its rapid germination phenotype. Three single spore clones of LH-EVOL were selected for further analysis. Each of the clones of LH-EVOL was able to germinate substantially faster than the parental AF293 strain in lung homogenate medium in the *in vitro* germination assay (Fig. 3A). Next, whole-genome sequencing of these three clones of LH-EVOL was conducted and compared with its parental AF293 strain. Whole-genome sequencing revealed conserved indel mutations in *Afu5g08390* across the three LH-EVOL clones (Fig. 3B). *Afu5g08390* encodes SskA which is a response regulator in the high-osmolarity glycerol (HOG) pathway (30). These indel mutations are predicted to insert premature stop codons in the *Afu5g08390* gene that may induce premature truncation before the recognition (REC) domain (Fig. 3C), a domain necessary for the signaling function of Ssk1, the SskA homologue, in yeast (31).

### LH-EVOL has a decreased SakA-dependent response under osmotic stress.

**FIG 4:**
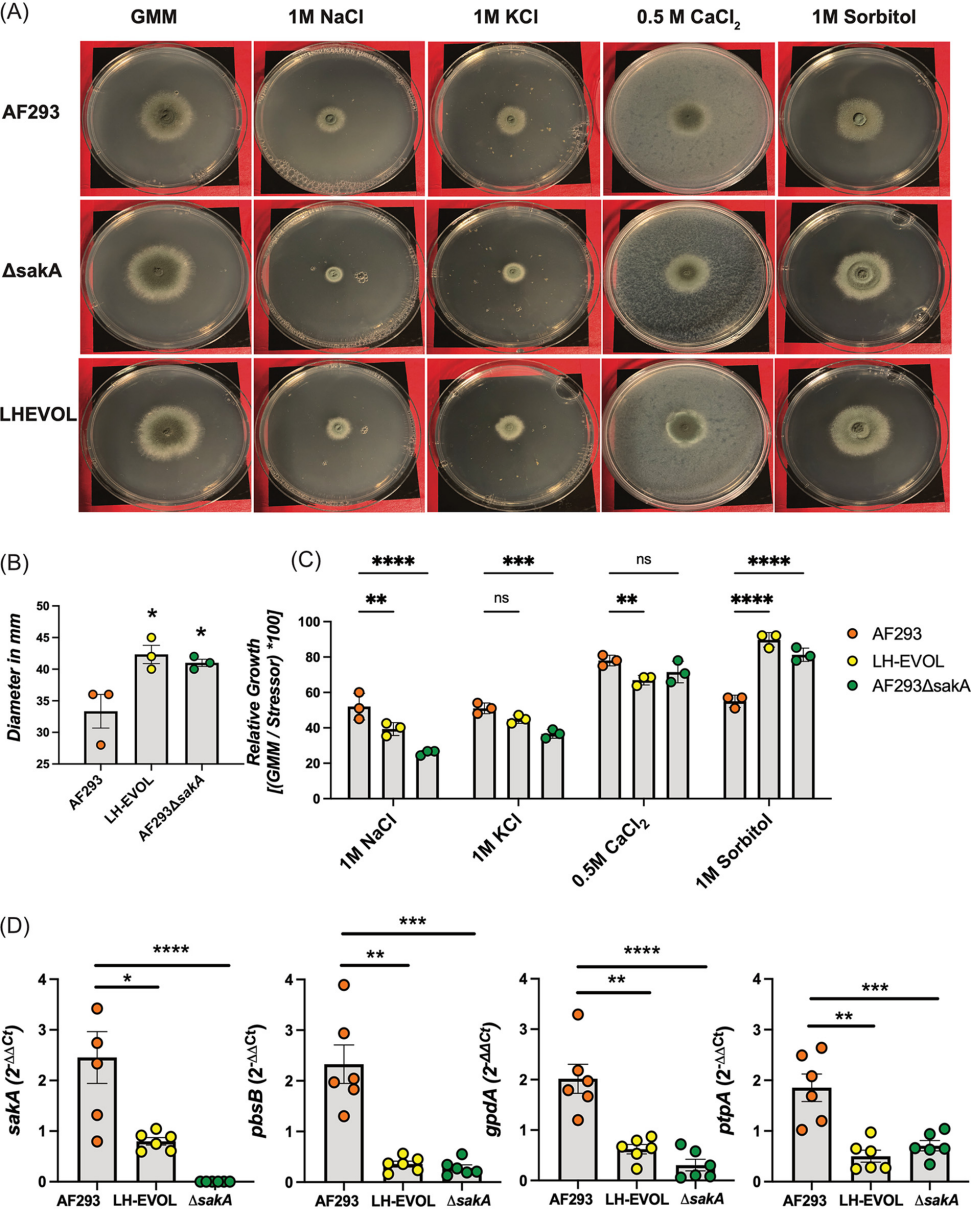
LH-EVOL strain demonstrates deficiencies in *sakA*-dependent osmotic stress response. (A) Strain AF293, AF293 Δ*sakA*, and LH-EVOL conidia were inoculated on GMM plates or GMM plates supplemented with 1 M NaCl, 1 M KCl, 0.5 M CaCl_2_, or 1 M sorbitol and incubated for 72 h at 37°C. (B) Diameter of colonies grown on GMM plates for 72 h were measured in millimeters. (C) Diameter of the colony was used to calculate the percentage of growth inhibition by each stressor as compared to its respective measurement on GMM. (D) A 30-h biofilm was transferred to fresh liquid GMM medium or fresh GMM medium supplemented with 1 M NaCl for 30 min at 37°C. Quantitative RT-PCR was used to measure the change in transcript production of *sakA* and *sakA*-dependent genes *pbsB*, *gpdA*, and *ptpA*. Threshold cycle (*Ct*) values were initially adjusted to *actA* for Δ*Ct*, then the GMM plus 1 M NaCl condition was compared to GMM condition for ΔΔ*Ct* values. Data are pooled results from two independent experiments. Statistical significance was assessed using a one-way ANOVA with Tukey’s test (B), two-way ANOVA (C), and one-way ANOVA with a Kruskal-Wallis test (D). *, *P* value of ≤0.05; **, *P* value of ≤0.01; ***, *P* value of ≤0.001; ****, *P* value of ≤0.0001; ns, not significant (*P* > 0.05).

SskA is a response regulator within the high-osmolarity glycerol pathway that drives the activation of the MAPK kinase kinase (MAPKKK) SskB, MAPKK PbsB, and MAPK SakA (32, 33). SakA is critical for maintaining homeostasis in A. fumigatus during osmotic stress (13, 34, 35). On the basis of the predicted truncation of SskA prior to its REC domain (Fig. 3C), we hypothesized that the LH-EVOL strain may exhibit a decreased SakA signaling response. To directly test this, strains AF293, AF293 Δ*sakA*, and LH-EVOL were inoculated onto GMM plates and GMM plates supplemented with 1 M NaCl, 1 M KCl, 0.5 M CaCl_2_, and 1 M sorbitol for 72 h at 37°C (Fig. 4A). LH-EVOL grown on GMM formed significantly larger colonies than AF293, similar to AF293 Δ*sakA* (Fig. 4B). Importantly, when LH-EVOL was grown on GMM plates supplemented with osmotic stressors, its growth was substantially altered compared with AF293, similar to the response of the AF293 Δ*sakA* mutant (Fig. 4C) (13). Specifically, both LH-EVOL and AF293 Δ*sakA* were significantly impaired in their ability to grow in the presence of 1 M NaCl, while growing significantly better in the presence of 1 M sorbitol.

Since strain LH-EVOL appears to have an altered response to osmotic stress similar to strain AF293 Δ*sakA*, we next explored the transcriptional responses of AF293, AF293 Δ*sakA*, and LH-EVOL biofilms to 1 M NaCl, which is known to drive SakA activation and signaling (13). Each strain was grown in liquid GMM medium for 30 h at 37°C before being transferred to either GMM or GMM supplemented with 1 M NaCl for 30 min. We chose to examine *sakA* expression itself, as well as SakA-dependent transcripts *ptpA*, *gpdA*, and *pbsB* which are highly upregulated in AF293 but not in AF293 Δ*sakA* following osmotic stress with 1 M NaCl (13, 36). Transcript levels of *sakA*, *ptpA*, *gpdA*, and *pbsB* were all increased to a significantly greater extent in parental strain AF293 as opposed to LH-EVOL and AF293 Δ*sakA* when exposed to 1 M NaCl (Fig. 4D). These data support the hypothesis that the allele of *Afu5g08390* (*sskA*) found in the LH-EVOL strain likely encodes an SskA truncation mutant with decreased signal transduction.

### Loss of *Afu5g08390* (*sskA*) in AF293 results in increased germination.

Since the indel mutations found within *Afu5g08390* (*sskA*) in the LH-EVOL strain are predicted to result in a premature truncation mutation (Fig. 3C), which resulted in decreased SakA-dependent transcriptional responses to osmotic stress (Fig. 4), we next tested whether the complete loss of SskA results in increased germination potential in strain AF293. To do this, we constructed an *sskA* null mutant using homologous recombination in the AF293 strain of A. fumigatus, as well as an ectopic complementation strain (see Fig. S1 in the supplemental material). When cultured on GMM plates, the AF293 Δ*sskA* strain had significantly greater radial growth than the parental AF293 strain, which was similar to what was observed with the AF293 Δ*sakA* and LH-EVOL strains (Fig. S2A). Next, to determine whether the AF293 Δ*sskA* strain had decreased SakA signaling, we explored the transcriptional responses of AF293, AF293 Δ*sakA*, and AF293 Δ*sskA* biofilms to 1 M NaCl. Each strain was cultured in GMM medium for 30 h at 37°C before being transferred to either GMM or GMM supplemented with 1 M NaCl for 30 min. Similar to previous results (Fig. 4D), transcript levels of *sakA*, *ptpA*, *gpdA*, and *pbsB* were all increased in AF293 relative to AF293 Δ*sakA* when exposed to 1 M NaCl (Fig. S2B to E). Importantly, AF293 Δ*sskA* failed to show elevated transcript levels of *sakA*, *ptpA*, *gpdA*, and *pbsB*, which supports the hypothesis that SskA-SakA signaling is decreased in the AF293 Δ*sskA* strains (Fig. S2B to E).

10.1128/msphere.00922-21.1 FIG S1 Construction and confirmation of A. fumigatus Δ*sskA* and Δ*sskA^RC^* strains. (A) Schematic of *sskA* gene replacement in A. fumigatus strain AF293.1 with expected band sizes following genomic DNA digestion with EcoRV. (B) Southern blot analysis of EcoRV-digested genomic DNA with a 1,088-bp digoxigenin-labeled probe reveals successful gene replacement of *sskA* with a single copy of *pyrG* in multiple transformants (KO.1, KO.2, KO.3, and KO.4). (C) Real-time quantitative PCR results for *sskA* mRNA levels in the wild type (WT) (AF293), Δ*sskA* (KO.4), and *sskA*^RC^ strains from 18-h liquid cultures. Download FIG S1, TIF file, 0.1 MB. Copyright © 2021 Kirkland et al. 2021 Kirkland et al. https://creativecommons.org/licenses/by/4.0/ This content is distributed under the terms of the Creative Commons Attribution 4.0 International license.

10.1128/msphere.00922-21.2 FIG S2 CEA10 and AF293 Δ*sskA* strains demonstrate deficiencies in *sakA*-dependent osmotic stress response. (A) Diameter of colonies grown on GMM for 72 h in millimeters. (B to E) A 30-h biofilm was transferred to fresh liquid GMM medium supplemented with 1 M NaCl for 30 min at 37°C. (B to E) Quantitative RT-PCR was used to measure the change in transcript production of *sakA* (B) and *sakA*-dependent genes *pbsB* (C), *ptpA* (D), and *gpdA* (E). *Ct* values were initially adjusted to *actA* for Δ*Ct*, then 1 M NaCl was compared to GMM for ΔΔ*Ct* values. Data are pooled of results from two independent experiments. Statistical significance was assessed using a one-way ANOVA with Tukey’s test (B) and Mann-Whitney test and one-way ANOVA with a Kruskal-Wallis test. (D) **, *P* value of ≤0.01; ***, *P* value of ≤0.001; ****, *P* value of ≤0.0001. Download FIG S2, TIF file, 1 MB. Copyright © 2021 Kirkland et al. 2021 Kirkland et al. https://creativecommons.org/licenses/by/4.0/ This content is distributed under the terms of the Creative Commons Attribution 4.0 International license.

**FIG 5:**
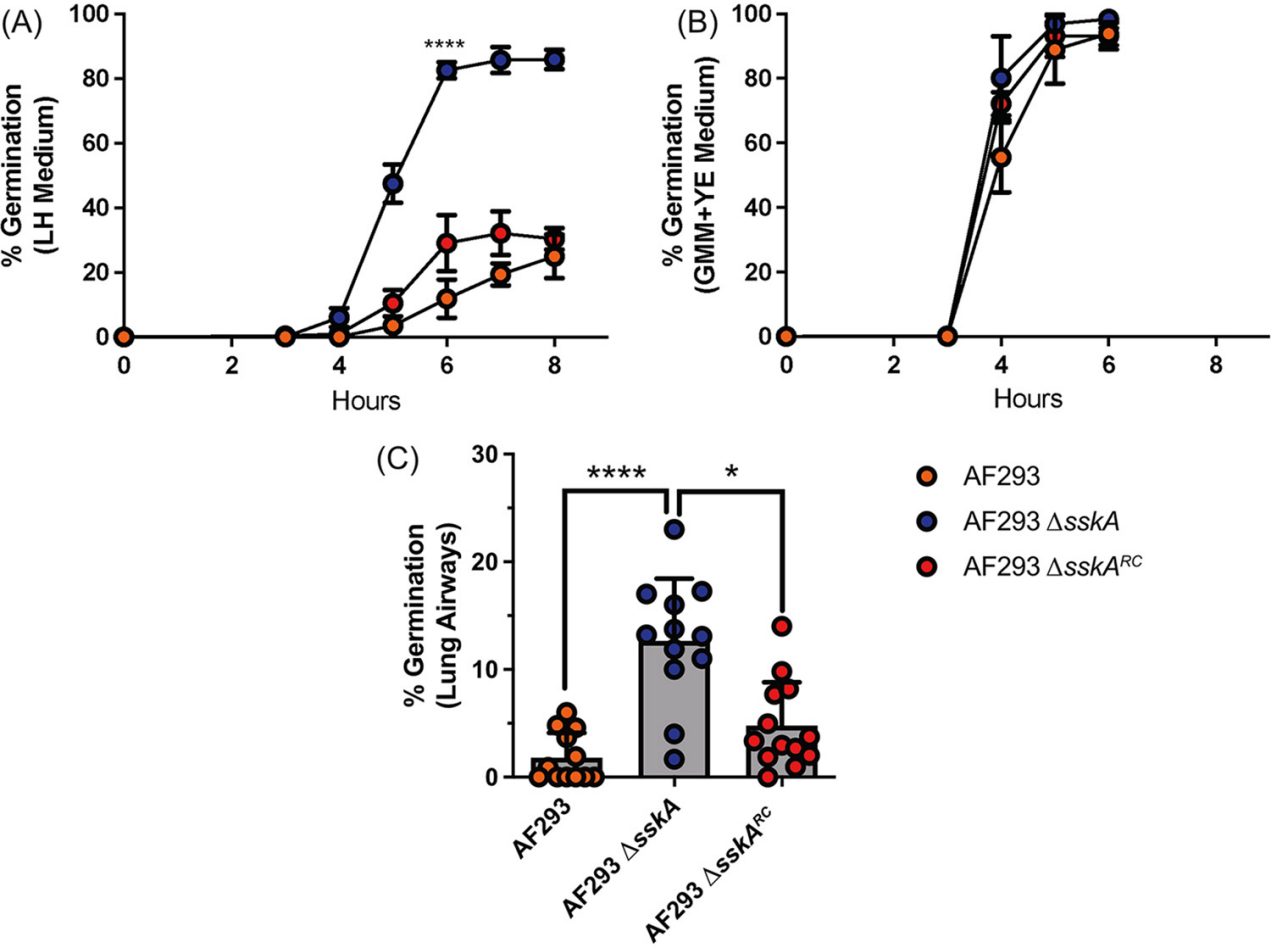
Loss of *sskA* in strain AF293 increases germination rate. (A and B) Conidial germination of AF293, AF293 Δ*sskA*, and AF293 Δ*sskA^RC^* strains was determined by wet mounts and microscopically counting the numbers of conidia and germlings in either lung homogenate medium (A) or 1% GMM supplemented with yeast extract (B). Data are representative of at least two independent experiments consisting of three biological replicates per group. Each symbol represents the group mean ± 1 standard error of the mean. (C) *In vivo* conidial germination in airways of C57BL/6J mice challenged with 4 × 10^7^ conidia of AF293, AF293 Δ*sskA*, and AF293 Δ*sskA^RC^* was assessed at 12 hpi. Mice were euthanized and BALF samples were collected to quantify germination in the airways. Data are pooled results from two independent experiments. Statistical significance was assessed using an unpaired *t* test (A) or one-way ANOVA with a Dunn posttest (C). *, *P* value of ≤0.05; ****, *P* value of ≤0.0001.

Next, to determine whether strain AF293 Δ*sskA* was able to germinate faster than AF293 or the complemented AF293 Δ*sskA^RC^* strain, we conducted an *in vitro* germination assay in lung homogenate medium. When AF293 Δ*sskA* was grown in lung homogenate medium, robust germination was observed compared to the parental strain or the complemented AF293 Δ*sskA^RC^* (Fig. 5A). In contrast, all three strains germinated at equivalent rates in nutrient-rich GMM supplemented with 0.5% yeast extract (GMM+YE) medium (Fig. 5B), demonstrating a lack of intrinsic changes to the overall germination potential of the AF293 Δ*sskA* strain. To determine whether AF293 Δ*sskA* also has an increased germination rate during initial airway infection of mice, C57BL/6J mice were inoculated intratracheally with either AF293, AF293 Δ*sskA*, and AF293 Δ*sskA^RC^*. Twelve hours postinoculation, AF293 Δ*sskA* had germinated to a significantly greater degree than either the AF293 or AF293 Δ*sskA^RC^* (Fig. 5C). Thus, loss of SskA signaling in AF293 strain of A. fumigatus results in enhanced germination potential within the lung microenvironment.

### Loss of the MAPKs SakA and MpkC results in increased germination.

**FIG 6:**
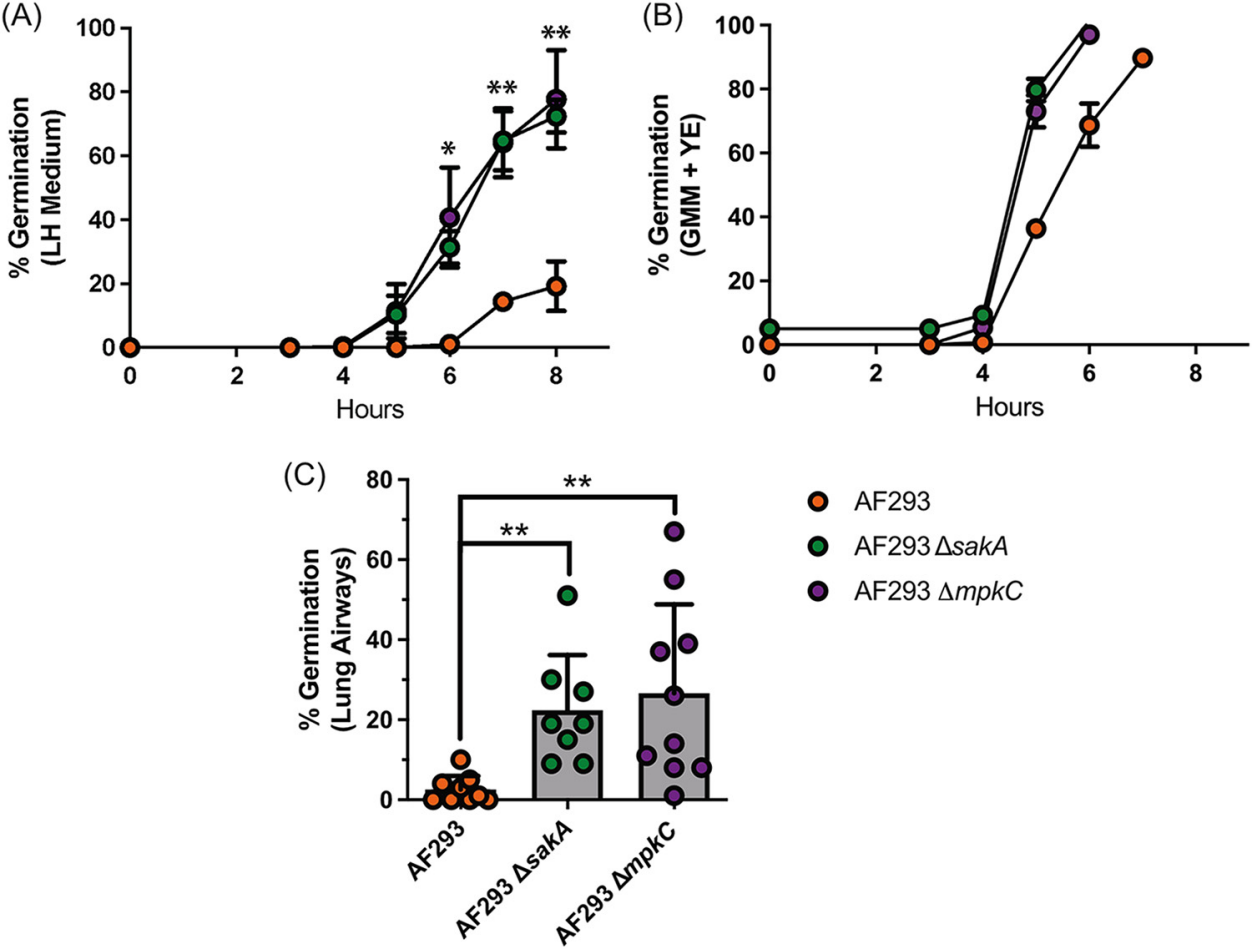
SakA and MpkC null mutants have increased conidial germination kinetics *in vitro* and *in vivo*. (A and B) Conidial germination of AF293, AF293 Δ*sakA*, and AF293 Δ*mpkC* strains was determined by wet mounts and microscopically counting the numbers of conidia and germlings in either lung homogenate medium (A) or 1% GMM supplemented with yeast extract (B). Data are representative of at least two independent experiments consisting of three biological replicates per group. Each symbol represents the group mean ± 1 standard error of the mean. (C) *In vivo* conidia germination in airways of C57BL/6J mice challenged with 4 × 10^7^ conidia of AF293, AF293 Δ*sakA*, and AF293 Δ*mpkC* was determined at 12 hpi. Mice were euthanized and BALF samples were collected to quantify germination in the airways. Data are pooled results from two independent experiments. Statistical significance was assessed using an unpaired *t* test (A) or one-way ANOVA with a Dunn posttest (C). **, *P* value of ≤0.01.

SakA associates with MpkC, a MAPK of the cell wall integrity pathway, in the nucleus (34, 37). Therefore, we wanted to test the roles of both the SakA and MpkC MAPK proteins in the regulation of germination of AF293 in the pulmonary environment. To determine whether SakA and MpkC regulate A. fumigatus germination, the rates of conidial germination of the AF293 Δ*sakA* and AF293 Δ*mpkC* mutant strains were assessed in lung homogenate medium. We found that germination indeed increased in both the AF293 Δ*sakA* and AF293 Δ*mpkC* strains relative to the parental strain (Fig. 6A). Additionally, the germination rate of both AF293 Δ*sakA* and AF293 Δ*mpkC* were similar to what we had observed for LH-EVOL and AF293 Δ*sskA* (Fig. 1B and Fig. 4A, respectively). In contrast, all strains have similar germination rates in GMM+YE medium, demonstrating that there were no global increases to the overall germination potential of these mutants (Fig. 6B). To determine whether AF293 Δ*sakA* and AF293 Δ*mpkC* also have increased germination rates during initial airway infection of mice, C57BL/6J mice were inoculated intratracheally with either AF293, AF293 Δ*sakA*, or AF293 Δ*mpkC*. Twelve hours postinoculation, both AF293 Δ*sakA* and AF293 Δ*mpkC* had germinated to a significantly greater degree than their parental strain (Fig. 6C). Thus, SskA signaling appears to converge on both the SakA and MpkC MAPKs in the AF293 strain of A. fumigatus to regulate germination within the lung microenvironment.

### Protein sequence analysis of the SakA signaling components in CEA10 identifies differences in TcsB and MpkC.

As the CEA10 strain of A. fumigatus is able to rapidly germinate both *in vitro* in lung homogenate medium and *in vivo* in murine lungs (4), we explored CEA10’s transcriptional response to 1 M NaCl in the biofilm transfer assay. Specifically, the CEA10 strain was cultured in liquid GMM for 30 h at 37°C before being transferred to either GMM or GMM supplemented with 1 M NaCl for 30 min. While strain AF293 had elevated transcript levels of *sakA*, *ptpA*, *gpdA*, and *pbsB*, strain CEA10 did not have increased levels of these transcript (Fig. S2). These data support the hypothesis that SskA-SakA signaling is altered in CEA10 relative to AF293.

Next, we compared the SskA amino acid sequences between strains AF293 and CEA10. By aligning the sequences with BLASTp, we found that the SskA protein sequences were 100% identical between AF293 and CEA10 (see Table S4 in the supplemental material). Next, we expanded our BLASTp analysis to compare the orthologs of all the known components of the SskA-SakA signaling pathway in the two strains. This analysis revealed that YpdA, SskB, PbsB, and SakA were also 100% identical between AF293 and CEA10 (Table S4). In contrast, BLASTp sequence alignment of the putative histidine kinase response regulator TcsB, homologue to Saccharomyces cerevisiae SLN1 and integral to the activation of Ssk1 in yeast (32, 38, 39), revealed a 13-amino-acid deletion (amino acids [AA] 709 to 721, XP_001481640.1) within the predicted ATPase (AA 696 to 863, XP_001481640.1) domain of the TcsB histidine kinase in CEA10 that is not present in AF293 (Fig. S3). Additionally, BLASTp sequence alignment of MpkC, which can interact with SakA signaling (34, 37, 40, 41), shows 4 amino acid changes (Table S4). Thus, the results of our protein sequence analysis suggest that changes in the SskA protein of CEA10 are likely not responsible for the increased germination rate of that strain, while changes in other proteins in the SakA signaling network, particularly TcsB, might be functionally different.

10.1128/msphere.00922-21.7 TABLE S4 GenBank accession numbers for protein sequences used in BLASTp analysis in nonredundant database. Download Table S4, DOCX file, 0.03 MB. Copyright © 2021 Kirkland et al. 2021 Kirkland et al. https://creativecommons.org/licenses/by/4.0/ This content is distributed under the terms of the Creative Commons Attribution 4.0 International license.

### SskA-SakA signaling limits A. fumigatus germination in decreased glucose conditions.

**FIG 7:**
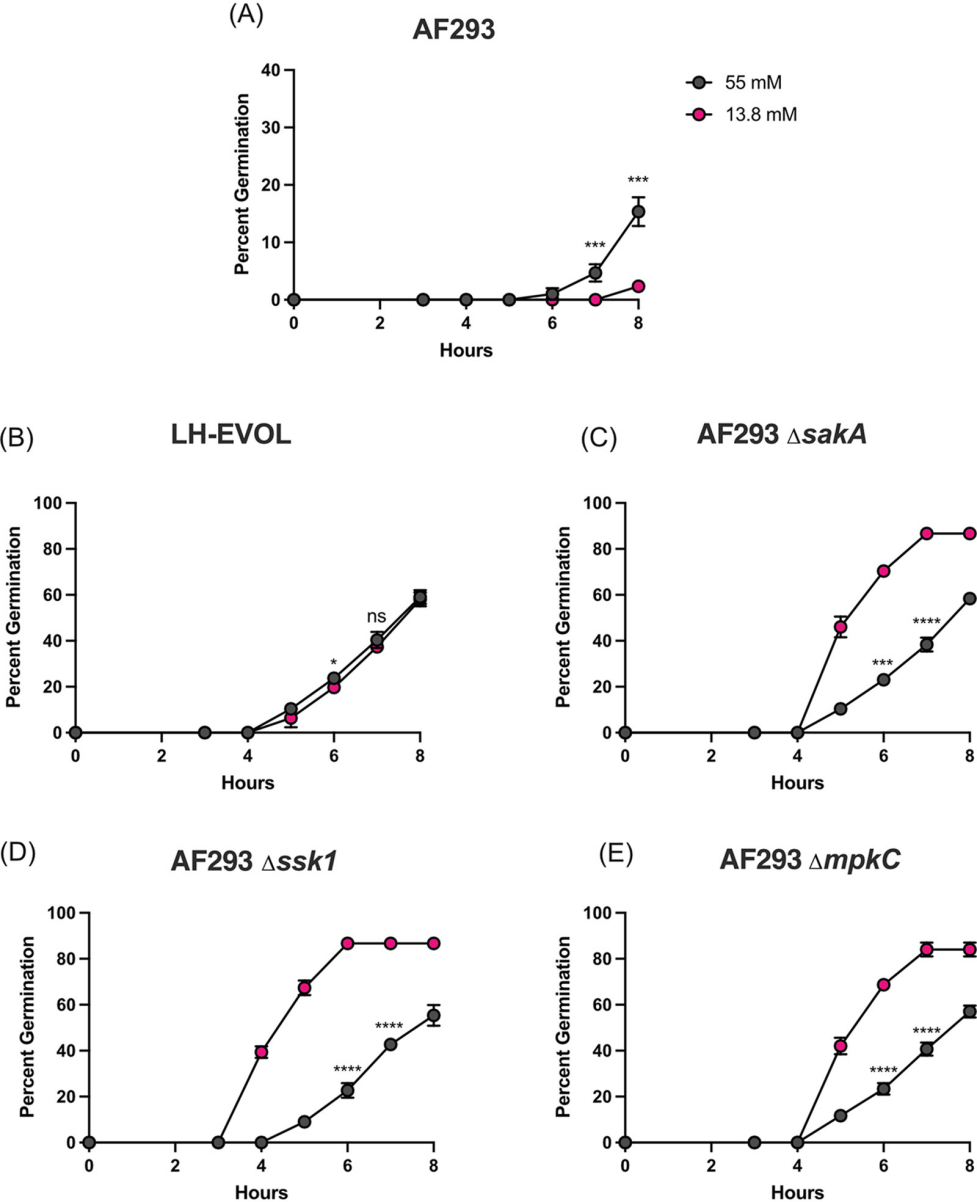
Increased germination rate of lung-adapted A. fumigatus strains in low glucose conditions. (A to E) Conidial germination of strains AF293 (A), LH-EVOL (B), AF293 Δ*sakA* (C), AF293 Δ*sskA*, (D) and AF293 Δ*mpkC* (E) was determined by wet mount and microscopically counting the numbers of conidia and germlings in either 1% GMM or 0.25% GMM medium. Statistical significance was assessed using an unpaired Student’s *t* test. *, *P* value of ≤0.05; ***, *P* value of ≤0.001; ****, *P* value of ≤0.0001; ns, not significant (*P* > 0.05).

The ASL which lines the airway epithelium, including the lungs, is a key component of respiratory homeostasis and facilitates mucociliary clearance of foreign particulates and pathogens. The glucose concentration of ASL is approximately 0.4 mM, substantially lower than typical *in vitro* culturing conditions. In yeast, glucose starvation can mediate activation of the stress MAPK Hog1 in an Ssk1-dependent manner (41). Consequently, we hypothesized that limited glucose availability may limit AF293 germination through SskA-SakA activation. To test this hypothesis, an *in vitro* germination assay using the AF293, LH-EVOL, AF293 Δ*sskA*, AF293 Δ*sakA*, and AF293 Δ*mpkC* strains was conducted in either 1% GMM (55.56 mM glucose) or 0.25% GMM (13.89 mM glucose). Interestingly, we found that while AF293 underwent limited germination in 1% GMM, it nearly completely failed to germinate in 0.25% GMM (Fig. 7A); however, the AF293 Δ*sskA* (Fig. 7C), AF293 Δ*sakA* (Fig. 7D), and AF293 Δ*mpkC* (Fig. 7E) strains underwent greater germination in 0.25% GMM than in 1% GMM. When we examined strain LH-EVOL, we found that the germination rate in both 1% GMM and 0.25% GMM were equivalent (Fig. 5B). Thus, it appears that in the absence of SskA-SakA signaling, there is an increased potential for A. fumigatus strain to germinate under low glucose conditions which would be found in the airways upon initial deposition.

## DISCUSSION

After entering the airways of a mammalian host, conidia are typically removed by mucociliary clearance or innate phagocytotic responses (1–3, 42). It has been proposed that fungal disease is driven by two types of virulence traits: (i) disease initiating factors, which are factors that either promote the initiation of infection or disease pathology; and (ii) disease progressing factors, which facilitate microbe persistence, host damage, and disease progression in an established infection microenvironment (43). Our previous work has demonstrated early fungal germination within the airway space is a disease initiating trait in a model of A. fumigatus-induced bronchopneumonia (4). However, the molecular mechanism(s) responsible for driving the increased fungal germination rate of certain A. fumigatus isolates remains elusive. In this study, we took an experimental serial passage approach to identify factors that may drive enhanced fungal germination. Through this approach we isolated a novel, lung-adapted strain, LH-EVOL, from the parental AF293 strain. The LH-EVOL strain showed increased germination *in vivo* in murine airways and *in vitro* in lung homogenate medium (Fig. 1) in addition to enhanced proinflammatory cytokine responses (Fig. 2).

Whole-genome sequencing of strain LH-EVOL revealed the presence of indel mutations in SskA that are predicted to result in the truncation of the SskA recognition (REC) domain (44) (Fig. 3), a domain necessary for SskA to dimerize with SskB to phosphorylate PbsB. In support of a predicted truncation of SskA’s REC domain, the LH-EVOL strain does not induce as strong a SakA-dependent response as measured by transcript abundance of SakA target genes during osmotic stress (Fig. 4); thus, the *sskA* allele identified in the LH-EVOL strain likely encodes an altered or nonfunctional SskA protein. However, one limitation of this study is that the function of the SskA protein found in LH-EVOL has not been directly determined. The prediction that the LH-EVOL allele of *sskA* results in a nonfunctional protein is, however, further supported by the finding that AF293 Δ*sskA* also germinates more rapidly than the parental strain *in vitro* in lung homogenate medium (Fig. 5A) and *in vivo* in the murine airways (Fig. 5C) which is what was observed in the LH-EVOL strain (Fig. 1). SskA is part of the SakA MAPK stress response pathway and has been shown to be important for SakA activation in the Afs35 strain of A. fumigatus (26). During germination of A. nidulans, SakA is dephosphorylated and exits the nucleus (37). Based on the location of the predicted premature stop codon due to an indel mutation found in the *sskA* gene of strain LH-EVOL (Fig. 3), it can be predicted that there would be less phosphorylated SakA in strains LH-EVOL and AF293 Δ*sskA*, making those strains poised to germinate quickly within the lung airways. This is further supported by our data demonstrating that AF293 Δ*sakA* also exhibits increased germination rates *in vitro* in lung homogenate medium (Fig. 6A) and *in vivo* in the airways (Fig. 6C). Interestingly, AF293 Δ*sakA* appears to undergo greater germination potential in the murine lungs than AF293 Δ*sskA*, although this was not compared directly. In S. cerevisiae, Hog1 signaling has two branches for its activation: Sln1 and Sho1 which are Ssk1 dependent and Ssk1 independent, respectively (45), which also seems to be the case in A. fumigatus (46). Our study examined only the role of the SskA-dependent branch in A. fumigatus germination, but future studies should examine the interplay of these two SakA signaling branches in regulating A. fumigatus germination.

As CEA10 is also a strain of A. fumigatus that can rapidly germinate in the lungs (4), we next examined the SskA-SakA signaling network in that strain. Interestingly, compared with strain AF293, strain CEA10 had a significantly weaker induction of SakA-dependent transcripts (see Fig. S2 in the supplemental material). However, the amino acid sequence identity of SskA and SakA wase 100% between strains AF293 and CEA10. Upstream of SskA, the histidine kinase TcsB, a homologue of SLN1 in yeast that is necessary for Ssk1 and HOG1 activation (32, 38, 39), in CEA10 has a 13-amino-acid deletion in the predicted ATPase domain (Fig. S3). This deletion could account for reduced expression of *pbsB*, *sakA*, and SakA-dependent genes in strain CEA10 during osmotic stress (Fig. S2), but this needs to be experimentally further explored. Thus, strains of A. fumigatus with decreased activity of SakA appear to have an increased potential for rapid germination and growth in murine lungs. Future studies should look at the relationship of A. fumigatus germination and the activity of specific SakA-dependent genes.

10.1128/msphere.00922-21.3 FIG S3 Amino acid alignment of sensory histidine kinase response regulator TcsB in strains AF293 and CEA10. (A) Amino acid sequence demonstrating deletion (709 to 721, XP_001481640.1) within the predicted ATPase (696 to 863, XP_001481640.1) in the histidine kinase domain in CEA10 compared to AF293. (B) Schematic of TcsB protein demonstrating the relative positions of amino acid deletion in CEA10. Download FIG S3, TIF file, 0.1 MB. Copyright © 2021 Kirkland et al. 2021 Kirkland et al. https://creativecommons.org/licenses/by/4.0/ This content is distributed under the terms of the Creative Commons Attribution 4.0 International license.

Within the pulmonary environment, the specific nutrient(s) driving A. fumigatus germination remains elusive. The airways of the lungs are a low-nutrient environment. Recent *in vivo* transcriptional analysis of the response of A. fumigatus in the murine airways demonstrates that A. fumigatus undergoes significant stress from iron, zinc, and nitrogen starvation (47). In our experiments, both the LH-EVOL strain and AF293-based SskA null mutant have an increased ability to germinate in 1% GMM compared to their parental AF293 strain (Fig. 5). Furthermore, the AF293-based SakA null mutant has an increased ability to germinate in 1% GMM (13). Interestingly, the ability of wild-type AF293 to germinate can be rescued by the supplementation of GMM with yeast extract (Fig. 5B) or complete medium containing yeast extract, peptone, and tryptone (13). This is significant because it has previously been shown that AF293 conidia are unable to utilize nitrate, the nitrogen source available in GMM media, as effectively as AF293 Δ*sakA* for growth in a SakA-dependent manner (13). Taken together, these data suggest that A. fumigatus may need to be able to effectively utilize nonpreferred nitrogen sources within the lung microenvironment to initiate germination, which will be explored in future studies.

Glucose levels in ASL from a healthy adult are approximately 0.4 mM (14). These levels are ∼10-fold reduced compared with the levels in plasma (14) and are thought to limit microbial growth, thus limiting infections (15). Surprisingly, we found that the strain LH-EVOL and AF293-based SskA, SakA, and MpkC null mutants were still able to germinate in 0.25% GMM, whereas the parental AF293 isolate was not. SakA has previously been shown to be directly involved in glucose uptake, glycogen and trehalose storage, and trehalose utilization by A. fumigatus (48). In addition, SakA-dependent glucose sensing modulates biofilm development (49). Furthermore, in Cryptococcus neoformans, Hog1 and protein kinase A (PKA) have been shown to be critical for adaptation to the glucose-limited environment of the lungs and glucose-replete environment of the brain, respectively (50). In low glucose conditions, Hog1 of C. neoformans becomes activated, resulting in translational repression through decreased ribosome biogenesis which is necessary for stress adaptation to the low-glucose environment of the lungs. In A. fumigatus, PKA signaling, regulated by SakA and MpkC (48), has been shown to be critical for growth in low-glucose environments and conidial germination (51). Considering these findings, our data would predict that the low-glucose environment of the lungs might similarly drive SakA activation in some A. fumigatus strains, such as AF293, limiting germination but increasing resistance against physiological stressors. In contrast, rapidly germinating strains may be more susceptible to osmotic and oxidative stressors in the host environment due to decreased SakA activity. This fits with the observation that strain CEA10 is more rapidly cleared than AF293 in a zebrafish model of infection (5). Consequently, SakA could be particularly important for A. fumigatus to adapt to the cystic fibrosis (CF) lung environment where there is increased oxidative stress (52) and ASL glucose concentrations can be 10 times higher than in healthy adults (16). Ross et al. demonstrated a distinct relationship between activation of the SakA pathway and adaptation by A. fumigatus to the lung environment in a CF patient (36). In that study, PbsB, an intermediary kinase between SskA and SakA, was found to have a missense mutation that resulted in increased SakA activity in response to osmotic stress (36). These data suggest a potentially important and distinct relationship between SakA activity in A. fumigatus in response to nutritional and osmotic stressors in the pulmonary environment, which may be critical to understanding disease initiation and progression within both acute and chronic models of aspergillosis.

Finally, our inflammatory profiling of pulmonary responses to both AF293 and LH-EVOL strains reinforces our knowledge that early germination in lung airways is a key driver of the distinct immunopathological phenotypes observed within different A. fumigatus isolates, where fast germinating isolates require an IL-1α-dependent host response to maintain host resistance (4) (Fig. 2C). Our inflammatory profiling also found that *Hif1a* mRNA was expressed to a greater extent following LH-EVOL challenge than strain AF293 (Table 2), fitting with our previous observation that hypoxia-inducible factor 1α (HIF1α)-dependent inflammatory responses are necessary for host resistance against the rapidly germinating CEA10 strain of A. fumigatus, but not AF293 (27).

Notably, we found a stronger IFN signaling signature following challenge with strain AF293 than with strain LH-EVOL (Fig. 2B). Recent work has demonstrated a critical role for both type I and type III IFNs in driving host resistance against A. fumigatus (53). Based on our NanoString analysis (Fig. 2), slow germinating strains of A. fumigatus may be more potent inducers of the type I and/or type III IFN response, which warrants further exploration. In support of this, human bronchial epithelial cells respond to resting conidia, rather than swollen conidia or hyphae, by enhanced secretion of IFN-β and CXCL10 (28). The expression of type I IFN (IFN-α and IFN-β) and CXCL10 can be induced by A. fumigatus double-stranded RNA (dsRNA) through both Toll-like receptor 3 (TLR3)-Trif and melanoma differentiation-associated protein 5 (MDA5)-mitochondrial antiviral signaling protein (MAVS)-dependent signaling (28, 54, 55). Overall, this suggests that the induction of IFN may occur in response to early development stages of A. fumigatus infection, while the robust proinflammatory response (e.g., IL-1α release) is driven by the later invasive hyphal stage. Differential host responses to other fungal infections over time have been previously described. For example, recent work examining the inflammatory response of vaginal epithelial cells to *Candida* spp. demonstrate an early, homogeneous type I IFN response to all *Candida* spp., but at later time points, the inflammatory response diverges in a species-specific and damage-dependent manner (56).

In conclusion, this study further emphasizes the strain-specific virulence, pathology, and inflammatory responses that occur during A. fumigatus infections. Our study also identifies a role for the loss of SakA MAPK signaling in enhancing conidial germination, particularly in low-glucose environments. This likely comes with the recompense of decreased resistance to osmotic and oxidative stress, which could be critical to disease progression during chronic fungal diseases (56). Thus, future studies must address the roles of these traits and pathways in both acute and chronic models of aspergillosis.

## MATERIALS AND METHODS

### Fungal strains and growth conditions.

All fungal strains used in this study are listed in Table S1 in the supplemental material. For conidial harvest, strains were cultured on 1% glucose minimal medium [GMM; 1% or 55.56 mM glucose, 6 g/liter NaNO_3_, 0.52 g/liter KCl, 0.52 g/liter MgSO_4_·7H_2_O, 1.52 g/liter KH_2_PO_4_ monobasic, 2.2 mg/liter ZnSO_4_·7H_2_O, 1.1 mg/liter H_3_BO_3_, 0.5 mg/liter MnCl_2_·4H_2_O, 0.5 mg/liter FeSO_4_·7H_2_O, 0.16 mg/liter CoCl_2_·5H_2_O, 0.16 mg/liter CuSO_4_·5H_2_O, 0.11 mg/liter (NH_4_)_6_Mo_7_O_24_·4H_2_O, and 5 mg/liter Na_4_EDTA; pH 6.5] agar plates for 3 days at 37°C, at which time conidia were collected by flooding plates with phosphate-buffered saline (PBS) with 0.01% Tween 80 and gently scraping mycelia using a cell scraper. Conidia were then filtered through sterile Miracloth, washed, and resuspended in PBS, and counted on a hemacytometer. Strains were stored in PBS and were used immediately for *in vitro* experiments or allowed to rest overnight for *in vivo* studies.

10.1128/msphere.00922-21.4 TABLE S1 Aspergillus fumigatus strains used in this study. Download Table S1, DOCX file, 0.03 MB. Copyright © 2021 Kirkland et al. 2021 Kirkland et al. https://creativecommons.org/licenses/by/4.0/ This content is distributed under the terms of the Creative Commons Attribution 4.0 International license.

### Serial passaging of AF293 through murine lung homogenate medium.

The wild-type AF293 strain of A. fumigatus was used for a serial passage experiment as it germinates slowly in the lung airways (6). Murine lung homogenate medium was made as previously described (6), where naive C57BL/6J lungs were crushed in 2 ml PBS and filtered through a 70-μm filter after which cellular debris was removed by centrifugation. The working concentration used was 1:4 in PBS and contains ∼35 μM glucose, which was determined using a glucose oxidase assay kit. Strain AF293 was cultured in lung homogenate medium for 8 h at 37°C with shaking at 350 rpm to induce germination. The fungal material was then collected and plated on 1% GMM agar plates and grown for 3 days at 37°C. The populations of conidia were then collected and catalogued. This process was repeated 13 times. Strain LH-EVOL was then grown under standard growth conditions prior to further analyses. Twenty individual clones of the LH-EVOL population were isolated to a single spore for further analysis in our lung homogenate germination assay.

### Whole-genome sequencing and variant identification.

To assess potential mutations within the LH-EVOL strain, whole-genome DNA sequencing was performed on three LH-EVOL clones (LH-EVOL clone 4, LH-EVOL clone 11, and LH-EVOL clone 12) with enhanced germination in lung homogenate medium. Genomic DNA was extracted from mycelia grown in petri plates using liquid 1% GMM supplemented with 0.5% yeast extract, following previously published methods (57). The DNA concentration was quantified using a Qubit 2.0 fluorometer (Invitrogen) and the manufacturer’s recommended Broad Range protocol. The genomic DNA sequencing was genomic sequencing libraries were prepared by SeqMatic (Fremont, CA) using Illumina TruSeq DNA kits and sequenced on a MiSeq Illumina sequencer in 2 × 250-bp format following the manufacturer’s recommendations for paired-end library construction and barcoding. The sequencing produced on average 1.1 million (1.1M) to 1.6M reads, which is equivalent to 300–440 Mb and 10× to 14× coverage of the A. fumigatus genome.

The DNA sequence reads for each strain were aligned to the AF293 reference genome downloaded from FungiDB v.39 (31, 58) with BWA v0.7.17 (59) and converted to BAM files using SAMtools v1.10 (60). Following best practices (61), the reads were marked for PCR and optical duplication using picard tools v2.18.3 (http://broadinstitute.github.io/picard). For variants identified near inferred indels, reads were realigned using RealignerTargetCreator and IndelRealigner in the Genome Analysis Toolkit GATK v3.7 (61). The single nucleotide polymorphisms (SNPs) and indels were genotyped relative to strain AF293 using HaplotypeCaller in GATK v4.1.1.0 (62). Filtering was accomplished with GATK’s SelectVariants with the following parameters: for SNPs, -window-size = 10, -QualByDept < 2.0, -MapQual < 40.0, -QScore < 100, -MapQualityRankSum < −12.5, -StrandOddsRatio > 3.0, -FisherStrandBias > 60.0, -ReadPosRankSum < −8.0; for indels, -window-size = 10, -QualByDepth < 2.0, -MapQualityRankSum < −12.5, -StrandOddsRatio > 4.0, -FisherStrandBias > 200.0, -ReadPosRank < −20.0, -InbreedingCoeff < −0.8. The genic overlap and consequence of the variants were classified with snpEff (63). Variants were transformed into tabular format from VCF format using script “snpEff_2_tab.py.”

### Aspergillus fumigatus pulmonary challenge model.

C57BL/6J mice (catalog no. 000664) were purchased from Jackson Laboratory at 7 to 8 weeks of age. *Il1a* ^−/−^ mice (64) were bred in-house at Dartmouth College. Animal studies were carried out in accordance with the recommendations in the *Guide for the Care and Use of Laboratory Animals* (65). The animal experimental protocol 00002168 was approved by the Institutional Animal Care and Use Committee (IACUC) at Dartmouth College. Mice were challenged with 4 × 10^7^ conidia in PBS intratracheally under isoflurane anesthesia in a volume of 100 μl. At the indicated experimental time points, mice were euthanized, and bronchoalveolar lavage fluid (BALF) was collected by washing the lungs with 2 ml of PBS containing 0.05 M EDTA. BALF was clarified by centrifugation and stored at −20°C until analysis. After centrifugation, the cellular component of the BALF was resuspended in 200 μl of PBS. BALF cells were subsequently spun onto glass slides using a Cytospin4 cytocentrifuge (Thermo Scientific) and stained with Diff-Quik stain set (Siemens) for quantification of fungal germination as previously described (4). The percent germination of each A. fumigatus strain was quantified by manual counting of 100 to 200 fungal conidia and germlings at ×100 magnification using a standard upright microscope. For histological analysis, lungs were filled with and stored in 10% buffered formalin phosphate for at least 24 h. Lungs were then embedded in paraffin, and lung sections were stained with Grocott’s methenamine silver (GMS) staining to assess fungal germination.

### Murine lung RNA preparation and NanoString analysis.

Animals were challenged with either PBS or strain AF293 or LH-EVOL as described above. Forty hours after inoculation, mice were euthanized, and whole lungs were removed for mRNA analysis. Lungs were placed in 2 ml of TRIzol and homogenized with a glass Dounce homogenizer, followed by treatment with chloroform to extract RNA according to the manufacturer’s instructions. After RNA was assessed for quality, 100 ng of RNA was used per reaction mixture using the nCounter Mouse PanCancer Immune Profiling Panel (NanoString). nSolver 4.0 software was used for background subtraction and normalization. nSolver Advanced Analysis 2.0 was used for quality control analysis and pathway analysis.

### Mutant A. fumigatus strain construction.

The Δ*sskA* strain was generated in the uracil/uridine auxotrophic strain AF293.1 through homologous recombination. The gene replacement construct was generated using overlap extension PCR as previously described (66) in which ∼1 kb of sequence 5′ to the start codon of *sskA* and ∼1 kb 3′ to the stop codon of *sskA* were amplified and fused to Aspergillus parasiticus orotidine 5′-monophosphate decarboxylase gene, *pyrG.* This *pyrG* fragment was amplified from pSD38.1 where *pyrG* has been blunt end ligated into pJET vector (catalog no. K1231; Thermo Scientific) with 5′ and 3′ linker sequences, 5′-ACCGGTCGCCTCAAACAATGCTCT-3′ and 5′-CGCATCAGTGCCTCCTCTCAGAC-3′, respectively (44). Primers for the amplification of this gene replacement construct are provided in Table S2. The transformation of strain AF293.1 to generate Δ*sskA* was carried out as previously described for the transformation of A. fumigatus protoplasts generated using lysing enzyme from Trichoderma harzianum (catalog no. L1412; Sigma) (67). Candidate Δ*sskA* strains were selected for restored prototrophy. Single spores were isolated from the candidate strains to generate clonal strains that were confirmed to be Δ*sskA* through PCR and Southern blot analyses. Southern blot analyses were performed as previously described using the digoxigenin-antidigoxigenin detection system (Roche Diagnostics) (67).

10.1128/msphere.00922-21.5 TABLE S2 Primers used to generate AF293 Δ*sskA* and AF293 Δ*sskA^RC^* strains. Download Table S2, DOCX file, 0.03 MB. Copyright © 2021 Kirkland et al. 2021 Kirkland et al. https://creativecommons.org/licenses/by/4.0/ This content is distributed under the terms of the Creative Commons Attribution 4.0 International license.

For the generation of the *sskA^RC^* construct, the pBluescript II KS(+) (Addgene) was utilized. The multiple cloning site was expanded, and the pyrithiamine resistance cassette was amplified from pPTR I (TaKaRa) to include XhoI (New England Biolabs) restriction sites at the 5′ and 3′ ends and introduced via the SalI site (pSD11). From strain AF293 genomic DNA, we amplified an ∼4-kb fragment that included ∼1 kb 5′ and ∼200 bp 3′ of *sskA* and introduced PacI and NotI (New England Biolabs) restriction sites at the 5′ and 3′ ends, respectively. The *sskA^RC^* construct, pSskA-R was confirmed through restriction digestion and Sanger sequencing. The construct was isolated (Zyppy Plasmid Miniprep; Zymo Research) and transformed into the Δ*sskA* fungal genome as described above. The *sskA^RC^* strain, which contained an ectopic insertion of the *sskA* wild-type allele, was confirmed with quantitative reverse transcription-PCR (qRT-PCR) as previously described (44). The housekeeping genes utilized were *tub2* and *actA.*

### Fungal germination assays.

*In vitro* fungal germination assays were conducted as previously described (6). Briefly, fungal strains were inoculated in either 1% GMM (55.56 mM glucose), 0.25% GMM (13.89 mM glucose), 1% GMM supplemented with 0.5% yeast extract (YE), or lung homogenate at a concentration of 1 × 10^7^ conidia/ml in 2 ml of medium in glass 20-ml disposable scintillation vials (VWR). Cultures were incubated at 37°C while shaking at 300 rpm. Starting at 4 h a wet mount was made every hour to count the number of germlings to conidia. Samples were vortexed with 1.0-mm beads to break up clumps before mounting, and fungal germination was quantified manually using the 40× lens objective of an upright VWR microscope. A minimum of 100 conidia and germlings for each sample were counted.

### Radial growth assay for testing response to osmotic stress.

GMM plates (1% glucose) were supplemented with 1 M NaCl, 1 M KCl, 0.5 M CaCl_2_, or 1 M sorbitol. A total of 1 × 10^5^ conidia were inoculated onto plates in 2-μl drops. The plates were incubated at 37°C for 72 h, at which point the diameter of the colony was measured. Inhibition was determined by normalizing each strain to their 72-h GMM growth.

### Fungal RNA extraction and qRT-PCR analysis for SakA-dependent transcripts.

For transcript analysis during growth during osmotic stress, 1 × 10^6^ conidia of AF293, AF293 Δ*sakA*, and LH-EVOL strains were grown in a six-well plate in 5 ml of GMM medium for 30 h at 37°C. Mycelia were transferred to a new six-well plate with either GMM or GMM supplemented with 1 M NaCl under sterile conditions and incubated for 30 min at 37°C. Mycelia were transferred to ZR BashingBead Lysis Tubes (0.1 and 0.5 mm; Zymo Research) and homogenized at 4°C using a bead beater for 4 min at 250 rpm. RNA was purified from lysate following the manufacturer’s protocol for Quick-RNA Fungal/Bacterial Miniprep kit (Zymo Research). cDNA was synthesized following the manufacturer’s protocol for QuantiTect reverse transcription kit (Qiagen) including genomic DNA (gDNA) removal. Quantitative PCR was performed using SsoAdvanced Universal SYBR Green Supermix according to the manufacturer’s directions and primers described in Table S3 using the thermocycler Bio-Rad C-1000 CFX96. Cycling parameters were according to the SsoAdvanced Universal SYBR Green Supermix manufacturer’s recommendation using 60°C as the annealing temperature. Transcripts were normalized to *actA* using the 2^−ΔΔCt^ method (68).

10.1128/msphere.00922-21.6 TABLE S3 Primers used for transcriptional analysis of SakA-dependent genes. Download Table S3, DOCX file, 0.03 MB. Copyright © 2021 Kirkland et al. 2021 Kirkland et al. https://creativecommons.org/licenses/by/4.0/ This content is distributed under the terms of the Creative Commons Attribution 4.0 International license.

### Protein sequence analysis.

Comparison of proteins of the SakA signaling network between strains CEA10 and AF293 was performed using the web Protein BLAST (BLASTp) platform from the U.S. National Library of Medicine, National Center for Biotechnology Information (https://blast.ncbi.nlm.nih.gov/Blast.cgi) searching the nonredundant protein sequence database. Protein sequences used for this analysis are listed in Table S4.

### Statistical analysis.

All graphs and statistical analyses were conducted using the GraphPad Prism 9 software. Statistical significance between experimental groups was determined using Student’s *t* test (comparison of two experimental groups normally distributed), a Mann-Whitney U test (comparison of two experimental groups that are not normally distributed), a one-way analysis of variance (ANOVA) using Dunn’s posttest (comparison of more than two experimental groups), or two-way ANOVA using Tukey’s posttest (comparison of more than two experimental groups with two variables) or Kruskal-Wallis posttest for non-Gaussian distributions.

### Data availability.

The genomic short reads for the four strains are deposited in NCBI SRA associated with BioProject accession no. PRJNA757188. Scripts for running the pipeline for analyses are available in GitHub (https://github.com/stajichlab/Afum_LH-EVOL).
