# Supplementary figures and images for: Host Lung Environment Limits Aspergillus fumigatus Germination through an SskA-Dependent Signaling Response
Source: mSphere. 2021 Dec 8;6(6):e00922-21. doi: 10.1128/msphere.00922-21 (PMC8653827; doi:10.1128/msphere.00922-21)

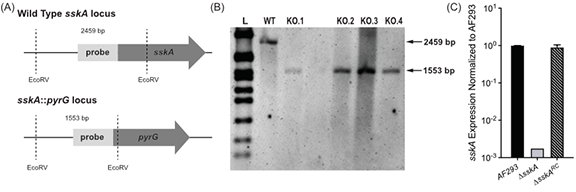

Supplement: FIG S1 [file msphere.00922-21-sf001.tif]

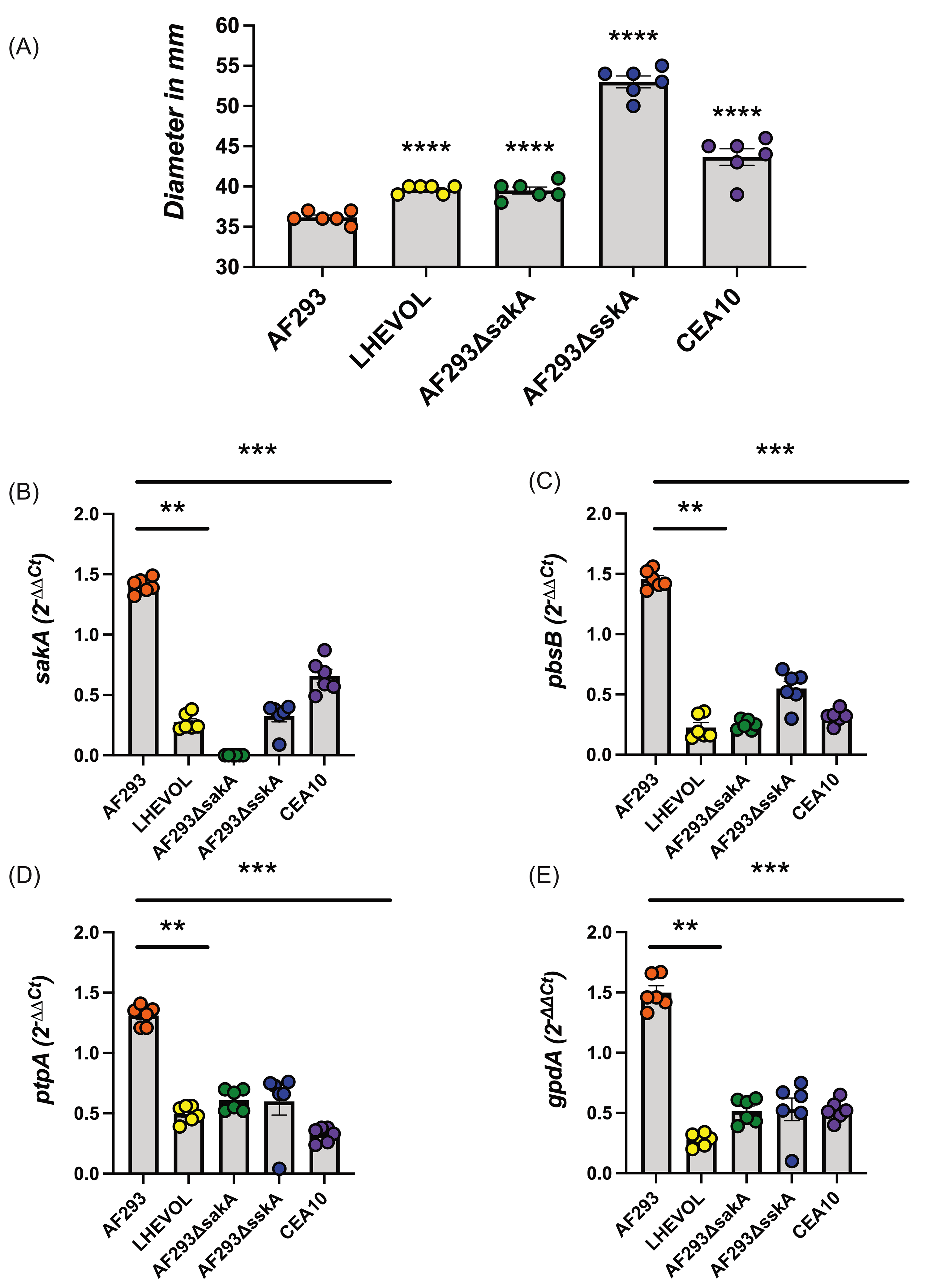

Supplement: FIG S2 [file msphere.00922-21-sf002.tif]

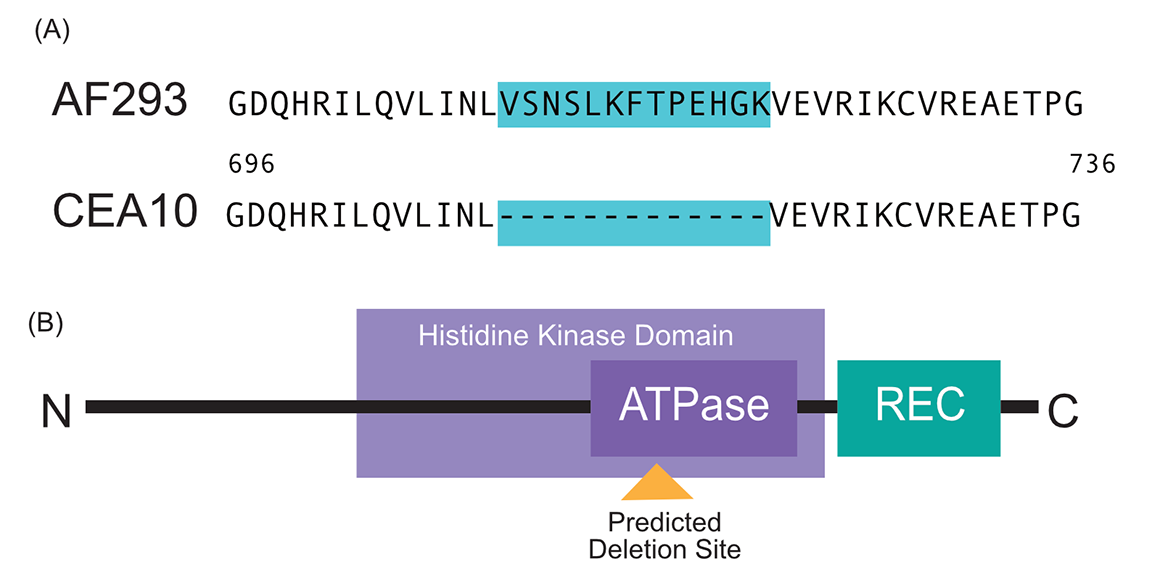

Supplement: FIG S3 [file msphere.00922-21-sf003.tif]
