# Supplementary material for: Host Lung Environment Limits Aspergillus fumigatus Germination through an SskA-Dependent Signaling Response
Source: mSphere. 2021 Dec 8;6(6):e00922-21. doi: 10.1128/msphere.00922-21 (PMC8653827; doi:10.1128/msphere.00922-21)
Supplement: TABLE S4 [file msphere.00922-21-st004.docx]

**Supplemental Table 4**. GenBank accession number for protein sequences used in BLASTp analysis in non-redundant database.

|  | **AF293** | **CEA10** | **Similarity (%)** |
| --- | --- | --- | --- |
| **TcsB** | XP_001481640.1 | EDP53731.1 | 97.87 |
| **YpdA** | XP_751798.2 | EDP50401.1 | 100 |
| **SskA** | XP_753797.1 | EDP51583.1 | 100 |
| **SskB** | XP_752459.1 | EDP56327.1 | 100 |
| **PbsB** | XP_752961.1 | EDP56828.1 | 100 |
| **SakA** | XP_752664.1 | EDP56531.1 | 100 |
| **MpkC** | XP_753727.2 | EDP51653.1 | 98.94 |
