# Supplementary material for: Host Lung Environment Limits Aspergillus fumigatus Germination through an SskA-Dependent Signaling Response
Source: mSphere. 2021 Dec 8;6(6):e00922-21. doi: 10.1128/msphere.00922-21 (PMC8653827; doi:10.1128/msphere.00922-21)
Supplement: TABLE S1 [file msphere.00922-21-st001.docx]

**SUPPLEMENTAL TABLE S1**. Strains used in this study. The strain identifiers, genotypes, and source information for all fungal strains included in this study.

| Strain ID | Genotype | Source |
| --- | --- | --- |
| AF293 | Reference strain | 1 |
| CEA10 | Reference strain | 2 |
| LH-EVOL | Experimentally evolved from AF293 | This study |
| AF293 Δ*sskA* | AF293 mutant lacking *sskA* | This study |
| AF293 Δ*sskA^RC^* | Ectopic complementation strain of the AF293 Δ*sskA* mutant | This study |
| AF293 Δ*sakA* | AF293 mutant lacking *sakA* | 3 |
| AF293 Δ*mpkC* | AF293 mutant lacking *mpkA* | 4 |

1. Nierman, W. C. *et al.* Genomic sequence of the pathogenic and allergenic filamentous fungus Aspergillus fumigatus. *Nature* **438**, 1151–1156 (2005).

2. Girardin, H., Latgé, J. P., Srikantha, T., Morrow, B. & Soll, D. R. Development of DNA probes for fingerprinting Aspergillus fumigatus. *J. Clin. Microbiol.* **31**, 1547–1554 (1993).

3. Xue, T., Nguyen, C. K., Romans, A. & May, G. S. A Mitogen-Activated Protein Kinase That Senses Nitrogen Regulates Conidial Germination and Growth in Aspergillus fumigatus. *Eukaryot. Cell* **3**, 557–560 (2004).

4. Reyes, G., Romans, A., Nguyen, C. K. & May, G. S. Novel Mitogen-Activated Protein Kinase MpkC of *Aspergillus fumigatus* Is Required for Utilization of Polyalcohol Sugars. *Eukaryot. Cell* **5**, 1934–1940 (2006).
