# Supplementary material for: Host Lung Environment Limits Aspergillus fumigatus Germination through an SskA-Dependent Signaling Response
Source: mSphere. 2021 Dec 8;6(6):e00922-21. doi: 10.1128/msphere.00922-21 (PMC8653827; doi:10.1128/msphere.00922-21)
Supplement: TABLE S2 [file msphere.00922-21-st002.docx]

**SUPPLEMENTAL TABLE 2.** Primers used to generate AF293 Δ*sskA* and AF293 Δ*sskA^RC^.*

| Strain ID/Purpose | Primers and Template DNA Information |
| --- | --- |
| *sskA^RC^* | Recon fragment from AF293 gDNA, PacI/NotI restriction site introduction |
|  | 5’-AAAAAATTAATTAATGACTGGGTGGAAATGTTTTTTCAGAGAG-3’  5’-AAAAAAGCGGCCGCTGGACGAATCAATAATGTGCGGGC-3’ |
|  | Real -time qPCR Primers for *sskA*  5’- CAACCGTTCCCGAATTG-3’  5’- GCTGGCTGGTGAATCT -3’ |
|  | Real-time qPCR Primers for *tub2*  5’- ATAATGTTCAGACCGCCCTCTGCT-3’  5’- GACGGATGTGGAATTGCCCACAAA-3’ |
|  | Real-time qPCR Primers for *actA*  5’- TCACTGCCCTTGCTCCCTCGTC-3’  5’- G GCACTTGCGGTGAACGATCGAA-3’ |
| ∆*sskA* | 5’ Flank Amplification from AF293 gDNA, includes overhang for *pyrG* fusion  5’-GCCTGGCGATAATGAGAGTCAG-3’  5’-AGA GCA TTG TTT GAG GCG ACC GGTCACAGTGCTTGACTGATAGCACAGC-3’ |
|  | 3’ Flank Amplification from AF293 gDNA, includes overhang for *pyrG* fusion  5’- CGCATCAGTGCCTCCTCTCAGACCCAGTTGCACTTTCTGCAAATCAGG-3’  5’- CCGCATCAAGGATGTCGTTGTCAG-3’ |
|  | *Aspergillus parasiticus pyrG* gene from pSD38.1  5’- ACCGGTCGCCTCAAACAATGCTCT-3’  5’- GTCTGAGAGGAGGCACTGATGCG-3’ |
|  | Fusion primers with 5’ fragment, 3’ fragment, and *pyrG* gene  5’- TGACTGGGTGGAAATGTTTTTTCAGAGAG-3’  5’- GCCCACTCTTTCTGACCCTGTTGA-3’ |
