# Supplementary material for: Host Lung Environment Limits Aspergillus fumigatus Germination through an SskA-Dependent Signaling Response
Source: mSphere. 2021 Dec 8;6(6):e00922-21. doi: 10.1128/msphere.00922-21 (PMC8653827; doi:10.1128/msphere.00922-21)
Supplement: TABLE S3 [file msphere.00922-21-st003.docx]

**Supplemental Table 3**. Primers used for transcriptional analysis of SakA-dependent genes.

| **Name** | **Forward Sequence 5’-3’** | | **Reverse Sequence 3’-5’** |
| --- | --- | --- | --- |
| *gpdA* | ACGAGATCAAGCAGGCCATC | | TCAGTGTAGCCGAGGATGTTC |
| *ptpA* | ACATTATGCCAGGGGACACA | | GATCATTGGCACCGCATCTT |
| *pbsB* | GCCTGGCCGCAAAAAGAATG | | AAGAGTCCCTGCCTTCGTGT |
| *sakA* | CCGTGAGCTGAAACTGTTGA | | GTCTGTCCCAAGGAGCTCTG |
| *actA* | AACTTGCGAGATAGCCTCCA | CCATGTCATCCCAGTTTGTG | |
